# A semantic relationship mining method among disorders, genes, and drugs from different biomedical datasets

**DOI:** 10.1186/s12911-020-01274-z

**Published:** 2020-12-14

**Authors:** Li Zhang, Jiamei Hu, Qianzhi Xu, Fang Li, Guozheng Rao, Cui Tao

**Affiliations:** 1 School of Economics and Management, Tianjin University of Science and Technology, Tianjin, 300457 China; 2 School of Biomedical Informatics, University of Texas Health Science Center at Houston, 7000 Fannin St Suite 600, Houston, TX 77030 USA; 3 College of Intelligence and Computing, Tianjin University, Tianjin, 300350 China; 4 Tianjin Key Laboratory of Cognitive Computing and Application, Tianjin, 300350 China

**Keywords:** Semantic relationship mining, Data integration, Disorder-gene-drug relationship

## Abstract

**Background:**

Semantic web technology has been applied widely in the biomedical informatics field. Large numbers of biomedical datasets are available online in the resource description framework (RDF) format. Semantic relationship mining among genes, disorders, and drugs is widely used in, for example, precision medicine and drug repositioning. However, most of the existing studies focused on a single dataset. It is not easy to find the most current relationships among disorder-gene-drug relationships since the relationships are distributed in heterogeneous datasets. How to mine their semantic relationships from different biomedical datasets is an important issue.

**Methods:**

First, a variety of biomedical datasets were converted into RDF triple data; then, multisource biomedical datasets were integrated into a storage system using a data integration algorithm. Second, nine query patterns among genes, disorders, and drugs from different biomedical datasets were designed. Third, the gene-disorder-drug semantic relationship mining algorithm is presented. This algorithm can query the relationships among various entities from different datasets.

**Results and conclusions:**

We focused on mining the putative and the most current disorder-gene-drug relationships about Parkinson’s disease (PD). The results demonstrate that our method has significant advantages in mining and integrating multisource heterogeneous biomedical datasets. Twenty-five new relationships among the genes, disorders, and drugs were mined from four different datasets. The query results showed that most of them came from different datasets. The precision of the method increased by 2.51% compared to that of the multisource linked open data fusion method presented in the 4th International Workshop on Semantics-Powered Data Mining and Analytics (SEPDA 2019). Moreover, the number of query results increased by 7.7%, and the number of correct queries increased by 9.5%.

## Background

Semantic web technology has been applied widely in the biomedical informatics field. The resource description framework (RDF) data model is commonly used to represent data in the database. A uniform resource identifier (URI) and character strings are used to represent different entities and the relationships between entities. These semantic datasets are published online and can be accessed via the HTTP protocol and are also known as linked open datasets [1]. For example, the Life Sciences dataset is one of the most important parts of Linked Open Data Cloud [2]. This database consists of 339 RDF datasets, including 234 BioPortal datasets, 35 Bio2RDF datasets, and 70 other datasets. Together, they contain over 30 billion semantic relationships. Furthermore, a vast number of semantic relationships has been extracted from biomedical literature databases with unstructured natural language texts (e.g., MEDLINE) [3, 4]. The other existing biomedical datasets include gene-related, disorder-related, and drug-related databases. For example, PharmGKB (https://www.pharmgkb.org) [5] is a database consisting of drugs, clinical guidelines, and gene-drug and gene-phenotype relationships. The UniProt (https://www.uniprot.org/) [6] database aims to provide comprehensive and high-quality resources on protein sequences and functional information. This database comprises UniProtKB, UniParc, UniRef, and the Proteomes dataset. The Kyoto Encyclopedia of Genes and Genomes (KEGG, https://www.genome.jp/kegg) database is a professional knowledge base for the biological interpretation of large-scale molecular datasets, such as genomic and metagenomic sequences [7]. The Semantic MEDLINE Database (SemMedDB) [3] (https://skr3.nlm.nih.gov/SemMedDB/index.html) is a repository of semantic predications (subject-predicate-object triples) from MEDLINE citations (titles and abstracts). This database currently contains approximately 98 million predictions from all PubMed citations (approximately 29.1 million citations, processed using MEDLINE BASELINE 2019) [8]. Over 3000 papers are added to MEDLINE every day. Therefore, new semantic relationships are constantly added to SemMedDB.

In recent decades, continuous effort has been directed to mining semantic relationships from biomedical literature text with machine learning approaches. Conditional random field (CRF) and support vector machines (SVM) have been used to mine relationships [9–11]. In [12], a new semisupervised learning method based on hidden Markov models is proposed to extract the disease candidate genes from the human genome. This method predicts genes by positive-unlabeled learning (PU-Learning). In [13], a verb-centric approach is proposed to extract relationships without a training dataset. In [14], Kilicoglu H et al. extend a rule-based, compositional approach that uses lexical and syntactic information to predict relationships.

An increasing number of graph-based mining techniques are being applied to characterize the semantic relations in semantic relation extraction tasks [15–17]. In [18], graph theory and natural language processing techniques are applied to construct a molecular interaction network to extract relationships automatically.

Deep learning models have been adapted to extract semantic relations for the biomedical domain. Moreover, this approach achieves high performance on different biomedical datasets [19]. For example, in [20], unsupervised deep learning models discovered 32% of new relationships not originally known in the UMLS. In [21], recurrent neural networks (RNNs) and convolutional neural networks (CNNs) are fused to learn the features. RNNs and CNNs are combined for high-quality biomedical relationship extraction.

However, various associations between different datasets are likely to exist. For example, a gene in KEGG could be associated with a gene in PharmGKB. Since KEGG stores data in a different way than PharmGKB, it is time-consuming and arduous to combine the two databases directly. Overall, gene, drug, and disorder information has been stored in different heterogeneous datasets. These different datasets contain essential pieces of information for the identification of potential disorder biomarkers. Heterogeneity and fragmentation of these biomedical datasets make it challenging to quickly obtain essential information regarding particular genes, drugs, and disorders of interest. Furthermore, searching these enormous datasets and integrating the findings across the heterogeneous sources is costly and complicated [22]. Drug repositioning is one of the urgent issues that requires semantic relationship mining among genes, disorders, and drugs from different biomedical datasets for precision medicine.

Generally, these datasets provide query access for users through an application programming interface. Querying the relationships among genes, drugs, and disorders has become a research topic of increasing interest. The research on linked datasets capitalizes on the storage, management, and querying of information and promotes in-depth data analysis and data mining [23]. Semantic relationship mining among genes, disorders, and drugs is widely used, for example, in precision medicine and drug repositioning. For example, semantic relationships among diseases, drugs, genes, and variants are used to automatically identify potential drugs for precision medicine in the Precision Medicine Knowledgebase (PreMedKB) [24]. The semantic relationships between any two or more entities are queried to obtain comprehensive information. The semantic relationships among genes, disorders, drugs, and other concepts in a knowledge base can also be exploited for prioritizing drug repurposing or repositioning [25–27]. Drug repositioning is a relatively inexpensive and fast alternative to the lengthy and financially onerous task of new drug development [28]. Semantic relationship mining between a drug and other molecules or entities can also be used for drug-related knowledge discovery [29] and cooccurring entities analysis [30]. However, because these datasets could be stored in different places and in different ways, with different data formats and inconsistent representations of the same entity, the power of data mining across multiple datasets is far from being realized.

In this paper, a semantic relationship mining method among genes, disorders, and drugs from different biomedical datasets is presented. Semantic relationship mining across different biomedical datasets was performed to address this problem.

Parkinson’s disease (PD) is a pervasive neurodegenerative disorder that affects approximately 6 million people worldwide. Genes play an essential role in the development of PD. Monogenic forms account for approximately 10% of all PD cases [31], while the other cases are multifactorial. An increasing number of PD loci have been identified [32]. We used PD as a case study and focused on mining the putative and most current disorder-gene-drug relationships of PD from four different biomedical datasets. We addressed some of the current challenges in the field, such as integration with different existing medical datasets and the exploitation of semantic relationship mining in real-case scenarios. This approach transcends the limitations of distributed heterogeneous data sources and results in more complete datasets in such a way that medical researchers can freely access multiple datasets across platforms. This study will impact future translational medical research.

## Methods

### Multisource data integration

**Fig. 1:**
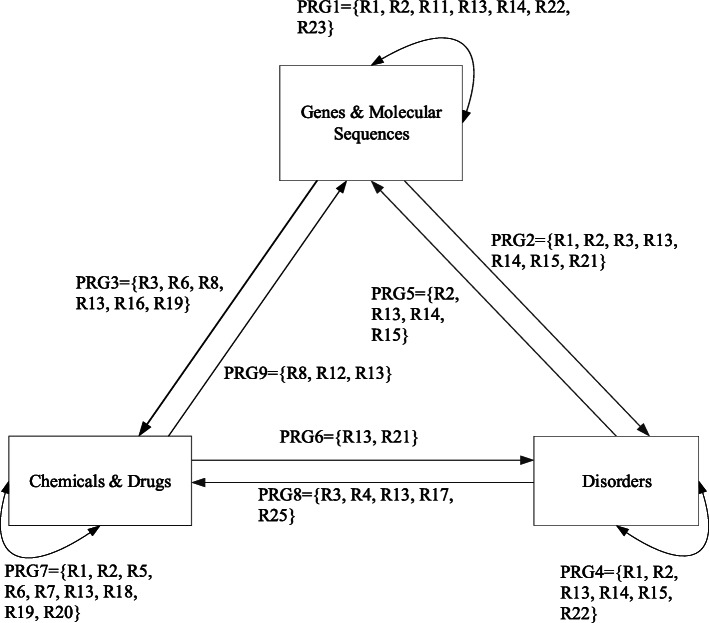
Gene-Disorder-Drug Relationships

The following life science datasets were studied in this paper: SemMedDB, KEGG, Uniprot, and PharmGKB. Different organizations publish these datasets. UMLS Metathesaurus was introduced to solve the morphology and polysemy problems. These datasets contain domain patterns for disorders (disorder), chemicals and drugs (drug) and genes and molecular sequences (gene). Figure 1 shows nine drug-disorder, gene-disorder, and drug-gene relationships.

Before mining, we converted the relational databases (including PharmGKB, KEGG, Uniprot, and SemMedDB) into the RDF data format using the D2R tool [33] to obtain the SemMedRDF, KEGGRDF, UniprotRDF and PharmGKBRDF datasets. We constructed Algorithm I to mine the semantic relationship types between SemMedRDF and other life science linked open data datasets. 
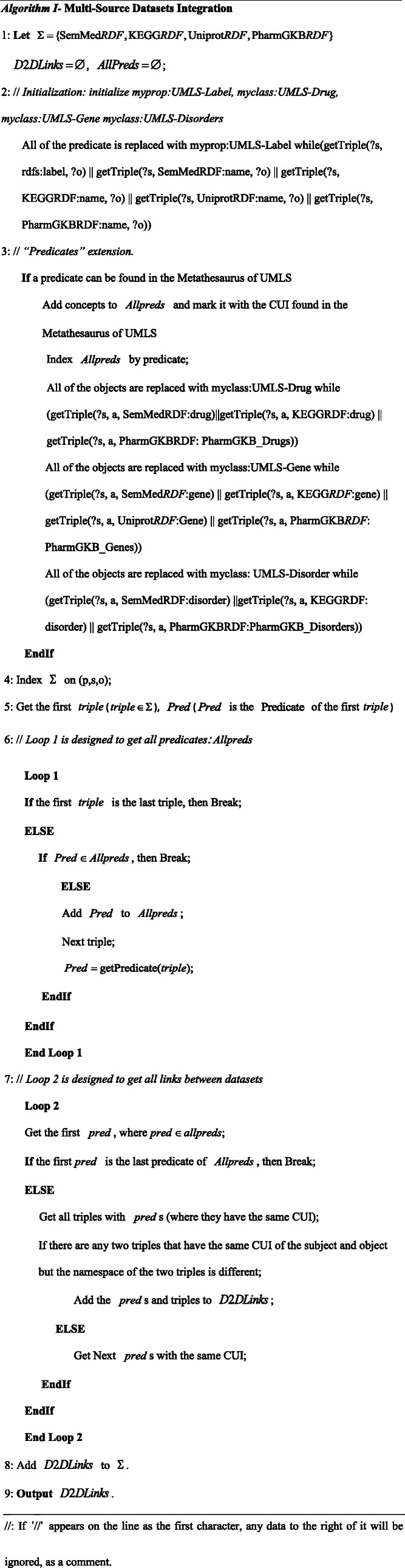


Algorithm *I* is described step by step as follows.

**Fig. 2:**
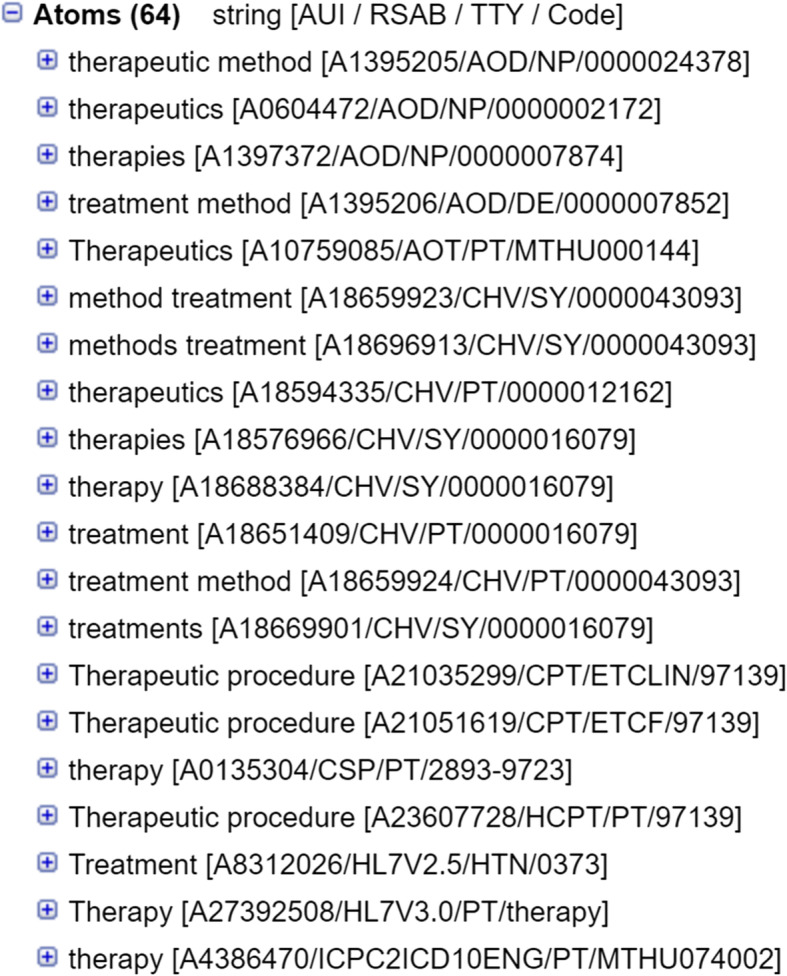
The search results extension of Predicate: “TREATS” in UMLS

The first step is variable initializations, where Σ is all data sets, including SemMedRDF, KEGGRDF, UniprotRDF and PharmGKBRDF. *Links* is a variable that saves a mined semantic relationship. Variable *AllPreds* stores the predicate of the datasets;

A compound index of *BMRDFs* is built on the predicate, subject, and object and will reduce the processing time;

The first triple is obtained from *BMRDFs*;

All of the predicates *Allpreds* are obtained from *BMRDFs*;

“Predicates” extension: If a predicate can be found in the Metathesaurus of UMLS, there will be several concepts with the same concept unique identifier (CUI), e.g., when searching the Predicate: “TREATS” in the Metathesaurus. The results are shown in Fig. 2. All of the concepts are added to *Allpreds* marking the CUI;

*Allpreds*is indexed on predicate;

The first *pred* of *Allpreds* is obtained;

If any two triples have the same CUI of the subject, predicate, and object while the namespace of the subject or object is different, this predicate will be one of the *Links*;

All of the *Links* will be added to *BMRDFs*. It will link the SemMedRDF to other biomedical datasets.

### Gene-disorder-drug semantic relationship mining

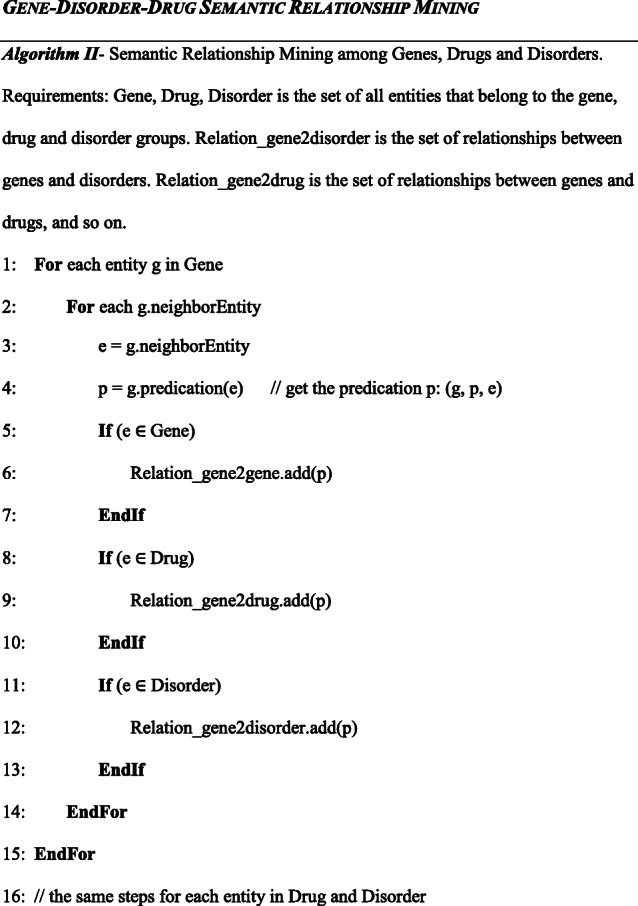



To fully understand the relationships among genes, disorders, and drugs, the following algorithm was designed to mine the attribute relationships among the three.

In Algorithm II, three entity sets are defined first: Gene, Drug, and Disorder. The relationships are defined among the three: the relational dataset from gene to disorder is called Relation_gene2disorder; the relational dataset from a gene to a drug is called Relation_gene2drug; other relational datasets can be named similarly. The algorithm to accomplish relationship querying is described as follows:

Traverse every entity in the Gene dataset;

Traverse the adjacent entity e of each entity and the predicate relationship p between the two;

If the adjacent entity e belongs to the element of Gene dataset, add the relationship p to Relation_gene2gene; if it belongs to the Drug dataset, add the relationship p to Relation_gene2drug; if it belongs to Disorder dataset, add the relationship p to Relation_gene2disorder.

Traverse each entity in the Drug and Disorder datasets to obtain the corresponding relational dataset.

### Query pattern design

Nine types of relational query patterns were designed based on the gene-drug-disorder relationships in Fig. 1. These query patterns are used in many research fields [25, 26, 34]. They are shown in Table 1.

**Table 1:** Query patterns

No.	Query pattern
Q1	Query all genes related to a specific gene
Q2	Query all disorders caused by a specific gene
Q3	Query all drugs targeting a specific gene
Q4	Query all disorders related to a specific disorder
Q5	Query all genes causing a specific disorder
Q6	Query all drugs treating a specific disorder
Q7	Query all drugs related to a specific drug
Q8	Query all disorders treated by a specific drug
Q9	Query all genes targeted by a specific drug

It is necessary to know the possible paths from a disorder to a drug to query the relevant drugs for a particular disorder, as shown in the relationship path in Fig. 1. For example, the algorithm designed for querying all drugs that treat a specific disorder is shown in Algorithm III. The remaining query processes can be performed in the same manner.

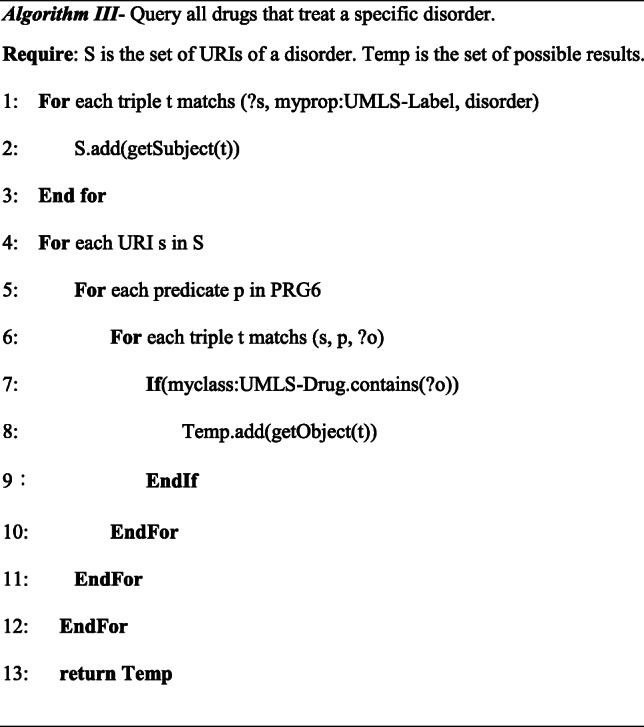



The algorithm to query all drugs that treat a specific disorder is described as follows:

Take the disorder name entered by the user as the object, and use the customized myprop: Label as the predicate to find the subject URI set S;

The relational set from disorder to drug analyzed in the previous section is the following: Traverse each URI in set S, and use each element in as predicate to query. The object set of the query is Temp;

Traverse temp to remove the elements that are not in myclass: Drug;

Output the remaining results in Temp.

Other algorithms for related queries are similar, except that the relational set changes.

## Experiments and results

### Experiment dataset

Overall, any biomedical datasets can be used to mine the semantic relationships among them. Here, we demonstrated how semantically integrated RDF datasets, extracted from structured biomedical databases or linked open data, can be used to automatically mine the semantic relationships among them. SemMedDB, KEGG, Uniprot, and PharmGKB were used in the experiment.

### Semantic relationship mining results

As shown in Table 2, 25 new relationships between the gene, disorder, and drug were mined from the SemMedRDF, KEGGRDF, UniprotRDF, and PharmGKBRDF datasets. As there are many relationships, the relationships in Fig. 1 were replaced by numbers, and each relationship set is represented by nine predicate relationship groups (PRG1-PRG9) in Table 3. For example, in row 2 of Table 3, the new relationships R1, R2, R11, R13, R14, R22, and R23 belong to PRG1. These relationships are also associated with the query patterns Q1. The new relationships can help us to mine more semantic relationships.

**Table 2:** Predicates and their corresponding numbers

No.	Predicates
R1	sem:coexists_with
R2	sem:interacts_with
R3	sem:causes
R4	sem:prevents
R5	sem:manifestation_of
R6	sem:affects
R7	sem:occurs_in
R8	sem:associated_with
R9	kegg:hasDisease
R10	kegg:hasDrug
R11	uniprot:externalLink
R12	pharmgkb: Related_Genes
R13	pharmgkb:associated
R14	sem:stimulates
R15	sem:inhibits
R16	sem:disrupts
R17	sem:treats
R18	sem:complicates
R19	sem:predisposes
R20	sem:augments
R21	sem:produces
R22	kegg:hasPathway
R23	kegg:hasGene
R24	pharmgkb: Related_Drugs
R25	pharmgkb:c2b2r_Related_Diseases

**Table 3:** Query patterns

No.	Related predicates	PRG (Predicates relationship group) No.
Q1	R1, R2, R11, R13, R14, R22, R23	PRG1
Q2	R1, R2, R3, R13, R14, R15, R21	PRG2
Q3	R3, R6, R8, R13, R16, R19	PRG3
Q4	R1, R2, R13, R14, R15, R22	PRG4
Q5	R2, R13, R14, R15	PRG5
Q6	R13, R21	PRG6
Q7	R1, R2, R5, R6, R7, R13, R18, R19, R20	PRG7
Q8	R3, R4, R13, R17, R25	PRG8
Q9	R8, R12, R13	PRG9

### Query results

1. Q1: Query all of the genes that are related to a specific gene, PARK2. There were 95 results (genes, proteins, and molecular sequences) related to PARK2, including PARK7, GCH1, PACRG, FBXW8, PINK1, and NBR1 (Table 4). Among them, 61 results were from SemMedDB, 23 results belonged to PharmGKB, and 11 results were from Uniprot.
2. Q2: Query all of the disorders caused by a specific gene, PARK2. There were 123 results (disorders) caused by PARK2. Some results were autosomal recessive juvenile Parkinson disease, leukemia, chronic myeloid leukemia, carcinoma of the large intestine, chronic obstructive airway disease, and chromosomal translocation. SemMedDB yielded 81 results, and another 42 results belonged to PharmGKB.
3. Q3: Query all drugs that target a specific gene, PARK2. There were 68 results (Chemicals & Drugs) that target PARK2. Some results were Cholesterol, multicatalytic endopeptidase complex, ubiquitin-protein ligase, FBXW8, and Reactive Oxygen Species. SemMedDB yielded 55 results, and another 13 results belonged to PharmGKB.
4. Q4: Query all disorders involved in a specific disorder, Parkinson’s. There were 66 results (disorders) involved in Parkinson’s. Some results were encephalitis, tremor, depressive disorder, hypokinesia, cognitive deficit, respiratory failure, equilibration disorder, and Lewy body disease. All of the results belonged to SemMedDB.
5. Q5: Query all of the genes that cause a specific disorder, Parkinson’s. There were 28 results (Genes, protein, and molecular sequences) involved in Parkinson’s. Some results were PARK1, PARK2, and CHCHD2. PharmGKB yielded 25 results, and another 3 results belonged to SemMedDB.
6. Q6: Query all of the drugs that treat a specific disorder, Parkinson’s. There were 51 results (Chemicals & Drugs) involved in Parkinson’s. Some results were dopamine, levodopa, dopamine transporter, and multicatalytic endopeptidase complex. SemMedDB yielded 40 results, and another 11 results belonged to PharmGKB.
7. Q7: Query all of the drugs involved in a specific drug, Levodopa. There were 79 results (Chemicals & Drugs) involved in Levodopa. Some results were Reserpine, Acetylcholine, Antipsychotic Agents, Monoamine Oxidase, Serotonin, and Isoproterenol. SemMedDB yielded 67 results, and another 12 results were from KEGG.
8. Q8: Query all of the disorders treated by a specific drug, Levodopa. There were 47 results (disorders) involved in Levodopa. Some results are Parkinson’s Disease, Seborrheic dermatitis, Hepatic Encephalopathy, Hepatic Coma, Hypotension, Secondary hyperprolactinemia due to prolactin-secreting tumor, Striatonigral Degeneration, nervous system disorder, and Hypokinesia. SemMedDB yielded 36 results, and another 11 results belonged to PharmGKB.
9. Q9: Query all of the genes that are targeted by a specific drug, Levodopa. There were 26 results (Genes, protein, and molecular sequences) involved in Levodopa. Some results were PARK1, PARK2, and CHCHD2. All of the results belonged to SemMedDB.

**Table 4:** Some genes related to PARK2

No.	Predicate	Object
1	<http://www4.wiwiss.fu-berlin.de/semdb/PREDICATE#COEXISTS_WITH>	<http://www4.wiwiss.fu-berlin.de/semdb/OBJECT_NAME#PARK7>
2	<http://www4.wiwiss.fu-berlin.de/semdb/PREDICATE#COEXISTS_WITH>	<http://www4.wiwiss.fu-berlin.de/semdb/OBJECT_NAME#GCH1>
3	<http://www4.wiwiss.fu-berlin.de/semdb/PREDICATE#COEXISTS_WITH>	<http://www4.wiwiss.fu-berlin.de/semdb/OBJECT_NAME#PACRGgene|PACRG>
4	<http://www4.wiwiss.fu-berlin.de/semdb/PREDICATE#COEXISTS_WITH>	<http://www4.wiwiss.fu-berlin.de/semdb/OBJECT_NAME#FBXW8>
5	<http://www4.wiwiss.fu-berlin.de/pharmgkb/ASSOCIATION#ASSOCIATED>	<http://www4.wiwiss.fu-berlin.de/pharmgkb/Entity2_NAME#PINK1>
…	…	…
95	<http://www4.wiwiss.fu-berlin.de/uniprot/EXTERNALLINK>	<http://www4.wiwiss.fu-berlin.de/uniprot#NBR1>

For the nine relationships between genes, disorders, and drugs, nine queries (Q1-Q9) were designed. Tables 5 and 6 record the source and respective proportions of each query result. To evaluate the results to improve the accuracy, we invited three professionals as domain experts to evaluate the query results. Two of these experts evaluated the results independently. The three experts provided their confidence levels (“Yes,” or “No”) in the query results. Each query result received the label “the correct query result” if it received more than two “Yes”. Otherwise, it was labeled “a false query result”. The analysis of the query results is shown in Tables 5 and 6: the column of “No” represents the nine queries. In the column of “(The number of correct queries results): (The number of queries results),”, for example, in Table 4, “48: 56” means that there were 56 query results from SemMedDB for Q1 in total. Forty-eight of them received the “correct results” label. The column “Precision” means that the “The number of correct query results” out of the total “The number of query results.” For example, in Table 4, “91.11” means that the “The number of correct query results” of Q1 was 91.11% (82/90).

**Table 5:** Analysis of the query results from [35]

No.	(The number of correct query results): (The number of query results)					Precision (%)
	SemMedDB	PharmGKB	KEGG	Uniprot	Total	
Q1	48: 56	23: 23	–	11:11	82: 90	91.11
Q2	56: 73	42: 42	–	–	98: 115	85.22
Q3	44: 52	13: 13	–	–	57: 65	87.69
Q4	54: 63	–	–	–	54: 63	85.71
Q5	–	25: 25	–	–	25: 25	100
Q6	29: 36	11: 11	–	–	40: 47	85.11
Q7	54: 61	–	12: 12	–	66: 73	90.41
Q8	25: 32	11: 11	–	–	36: 43	83.72
Q9	19: 23	–	–	–	19: 23	82.61
Total	329: 396	125: 125	12: 12	11: 11	477: 544	87.68

**Table 6:** Analysis of the query results from this paper

No.	(The number of correct query results): (The number of query results)					Precision (%)
	SemMedDB	PharmGKB	KEGG	Uniprot	Total	
Q1	53: 61	23: 23	–	11:11	87: 95	91.58
Q2	67: 81	42: 42	–	–	109: 123	88.62
Q3	48: 55	13: 13	–	–	61: 68	89.71
Q4	58: 66	–	–	–	58: 66	87.88
Q5	2: 3	25: 25	–	–	27: 28	96.43
Q6	34: 40	11: 11	–	–	45: 51	88.24
Q7	60: 67	–	12: 12	–	72: 79	91.14
Q8	31: 36	11: 11	–	–	42: 47	89.36
Q9	23: 26	–	–	–	23: 26	88.46
Total	376: 435	125: 125	12: 12	11: 11	524: 583	89.88

In Tables 5 and 6, the results are mainly from SemMedDB and PharmGKB. Furthermore, some of the results are from KEGG and Uniprot. The precision of PharmGKB, KEGG, and Uniprot was 100%. The precision of SemMedDB using the method in the paper published in the ISWC SEPDA 2019 workshop [35] was 83.08% (329: 396). The precision of SemMedDB using the method in this paper was 86.44% (376: 435), which was an increase of 4.04%.

The precision of the method published in the ISWC SEPDA 2019 workshop [35] was 87.68% (477/544). The precision of the method presented in this paper was 89.88% (524/583). The precision increased by 2.51%. Furthermore, the number of query results increased by 7.7% ((583–544)/583), and the number of correct query results increased by 9.5% ((524–477)/524). That means that the method in this paper can help mine more results with increased precision.

## Discussion

### Strengths

It is crucial to integrate SemMedDB with other databases in this method. SemMedDB is a database of semantic predictions (subject-predicate-object triples) from MEDLINE citations (titles and abstracts). SemMedDB currently contains approximately 98 million predictions from all PubMed citations (approximately 29.1 million citations, processed using MEDLINE BASELINE 2019) [8]. Over 3000 papers are added to MEDLINE every day. Therefore, new semantic relationships are added continuously to SemMedDB. The latest relationships can help to discover new relationships for related research. Some potential recommended drugs reported in the recent literature for PD have been found in the preliminary step work on drug repositioning based on this method.

In this paper, the semantic relationship mining method is used to explore interesting, hidden, or previously unknown biomedical relationships. Twenty-five new relationships are extracted in the verification experiment. It helps to improve the results with quantity and quality. Furthermore, interesting, hidden, or previously unknown biomedical relationships can help to detect the potential relationships between drugs and diseases [20, 36].

The nine types of common query patterns are proposed in the baseline method. This approach covers all semantic relationships between genes, disorders and drugs. Compared with the other models, our method can be extended to be used in more applications without a training dataset. Moreover, the method can also meet the requirements of processing large-scale data without high computational cost. The processing time increases with the size of the data linearly. It is more effective than the machine learning method, such as SemRep. In SemMedDB, the weighted average precision of the predictions is based on the number of predictions evaluated, which was approximately 0.79 [37–40]. In this paper, we used the approach in [34] to extract high-quality triples from SemMedDB. The precision increased by 2.27%.

### Limitations and future effort

Since the fact that the quality of the datasets will affect the semantic relationship mining, the method has some limitations: (1) The quality of the SemMedDB should be improved in future research. (2) The quality of the other datasets depends on their creators. Thus, high-quality datasets will be selected carefully. Alternatively, we will try our best to improve the quality of the datasets selected. (3) Currently, mining semantic relationships among genes, disorders, and drugs from different biomedical datasets is the first step for precision medicine and drug repositioning. It would be desirable to mine repositioning drugs based on semantic relationships for more disorders, such as PD, Alzheimer’s Disease, cancer.

## Conclusions

In this paper, a semantic relationship mining method among genes, disorders, and drugs was developed. In this method, data from various biomedical datasets were first converted into RDF triples and then integrated into a system for querying nine types of common query patterns. We focused on mining the putative and latest gene-disorder-drug relationships about PD. The experiment was conducted on four different datasets. The results showed that our method has significant advantages in integrating multisource heterogeneous biomedical data. Twenty-five new relationships among genes, disorder, and drugs were identified, and most of them came from different datasets. Moreover, the precision of our method increased by 2.51%. The number of query results increased by 7.7%, and the number of correct queries increased by 9.5%. These findings demonstrate that our method is robust and reliable in mining important gene-disorder-drug relationships.

## Data Availability

The PharmGKB is available at https://www.pharmgkb.org. The UniProt is available at https://www.uniprot.org. The KEGG is available at https://www.genome.jp/kegg. The SemMedDB is available at https://skr3.nlm.nih.gov/SemMedDB. The query results are available from the corresponding author upon request.

## Abbreviations

CUI: Concept unique identifier
KEGG: the Kyoto Encyclopedia of Genes and Genomes
PD: Parkinson’s disease
RDF: Resource description framework
SemMedDB: Semantic MEDLINE Database
URI: Uniform resource identifier
